# Evaluation of the Antifungal Activity of the Polyphenol Formulation *Viroelixir* Against *Candida albicans*

**DOI:** 10.3390/antibiotics15040420

**Published:** 2026-04-21

**Authors:** Manal Dahdah, Yasmine Ettouil, Hawraa Issa, Latifa Koussih, Mikhlid H. Almutairi, Mahmoud Rouabhia, Abdelhabib Semlali

**Affiliations:** 1 GREB Research Group, Faculty of Dentistry, Laval University, Québec, QC G1V 0A6, Canada; manal.dahdah.1@ulaval.ca (M.D.); yasmine.et-touil.1@ulaval.ca (Y.E.); mahmoud.rouabhia@fmd.ulaval.ca (M.R.); 2 DREAM-CV Lab, Centre for Outcomes Research and Evaluation, Research Institute of the McGill University Health Centre, Montreal, QC H4A 3J1, Canada; hawraa.issa.1@ulaval.ca; 3 Département des Sciences Expérimentales, Université de Saint-Boniface, Winnipeg, MB R2H 3B8, Canada; lkoussih@ustboniface.ca; 4 Zoology Department, College of Science, King Saud University, P.O. Box 2455, Riyadh 11451, Saudi Arabia; malmutairi@ksu.edu.sa

**Keywords:** *Candida albicans*, *Viroelixir*, antifungal activity, virulence, adhesion, SAPs expression, cytokines, TLR4-NF-κB pathway

## Abstract

*Candida albicans* (*C. albicans*) is an opportunistic fungal pathogen capable of causing a wide range of infections, including mucosal and systemic candidiasis. In the oral cavity, fungi represent a minor component of the microbiome but can significantly contribute to morbidity, particularly under conditions of dysbiosis or immunosuppression. Treatment remains challenging due to increasing multidrug resistance. This study investigates the in vitro antifungal potential of *Viroelixir*, a standardized polyphenol blend derived from green tea and pomegranate and enriched in catechins (including epigallocatechin gallate, EGCG), ellagitannins (notably punicalagin), ellagic acid, and flavonoids, with particular focus on its potential anti-virulence mechanisms. Methods: The effect of *Viroelixir* on *C. albicans* growth was assessed using MTT assay, optical density measurements, colony formation, carbohydrate quantification, and pH variation analysis. Biofilm formation, morphological transition, ROS production, necrosis, virulence gene expression, adhesion, and host immune responses were also evaluated. Results: *Viroelixir* significantly inhibited *C. albicans* growth and reduced colony formation compared with untreated controls. The formulation also inhibited biofilm formation and markedly reduced pseudohyphal development, reaching up to 94% reduction under specific treatment conditions. Flow cytometry analysis showed an increase in dead fungal cells, reaching approximately 88% following exposure to *Viroelixir* at the highest tested concentration. In addition, *Viroelixir* reduced the transcript levels of several virulence-associated genes, including SAP1–SAP9 and EAP1. In epithelial cell co-culture models, pre-treatment of *C. albicans* with *Viroelixir* reduced fungal adhesion and attenuated epithelial inflammatory responses, including IL-6, IL-8, and hBD-2 production, and was associated with reduced activation of the TLR4-NF-κB signaling pathway. Conclusions: These findings suggest that the antifungal and anti-virulence effects observed may be associated with the polyphenolic compounds present in the *Viroelixir* formulation, highlighting its potential as a promising in vitro antifungal candidate against *C. albicans*.

## 1. Introduction

A diverse microflora, consisting of over 700 species of bacteria, viruses, fungi, and protozoa, inhabits the human oral cavity [1]. Although fungi represent a relatively small proportion of the human microbiome, opportunistic fungal pathogens can cause a wide range of mucosal and systemic infections, particularly in immunocompromised individuals [2,3]. *Candida* species are responsible for the majority of human fungal infections, with *C. albicans* being the most frequently isolated species in cases of oral candidiasis [4,5]. While *C. albicans* commonly exists as a commensal organism in the oral, gastrointestinal, and genital tracts of many healthy individuals, under certain conditions it can cause superficial infections such as oral candidiasis or progress to invasive systemic infections [6,7]. Chronic infections and inflammation have also been implicated in carcinogenesis [8,9], and emerging evidence suggests that *C. albicans* may contribute to oral carcinogenesis through mechanisms including chronic inflammation, toxin production, and induction of pro-inflammatory cytokines [10,11,12,13,14].

The ability of *C. albicans* to undergo morphological transition from yeast to hyphae holds significant importance for establishing invasive disease by traversing mucosal barriers and entering deep tissues [6]. Another important factor influencing the virulence of *C. albicans* and ensuring resistance to antifungal agents is its ability to form biofilm on abiotic or biotic surfaces [7,15]. The transition from individual growth to growth within a biofilm, accompanied by a complex modification of phenotypic characteristics, is facilitated by changes in virulence gene expression [16]. Notably, hydrolytic enzymes and adhesins play essential roles in *C. albicans* pathogenicity. For example, epithelial adhesin protein 1 (EAP1) is involved in adhesion to human epithelial cells [17]. While secreted aspartyl proteases (SAPs) family members facilitate cellular digestion to support nutrient acquisition, tissue invasion, and immune evasion [18]. *C. albicans* initially interacts with epithelial cells, which play a key role in defending the body against various pathogens and serve as a barrier against oral candidiasis. This fungus is recognized by the Toll-like receptors (TLRs), such as TLR2 and TLR4, which trigger the production of pro-inflammatory cytokines and antimicrobial peptides in diverse cell types, including epithelial cells [19,20,21]. Specifically, β-defensins 2 and 3 demonstrate antifungal efficacy against *C. albicans* [22,23]. In response to infection, TLRs effects are mainly mediated by PKC, p38 MAPK, and NF-κB signaling pathways [24].

Current guidelines for the management of fungal infections are based on polyenes such as *Amphotericin B* (*Ampho-B*) (5 µg/mL), azoles, and echinocandins [25]. These antifungals function by targeting distinct fungal components: polyenes destroy the fungal cell membrane, azoles hinder ergosterol production, and echinocandins obstruct cell wall synthesis [26]. Despite their tendency to cause hepatotoxicity, ranging from asymptomatic liver abnormalities to very severe hepatocellular injury, causing fulminant liver failure [27], the polyenes class of antifungals are also costly. Another growing concern, associated with reduced treatment efficacy and the increasing prevalence of opportunistic and systemic fungal infections, is microbial resistance, which primarily affects immunocompromised individuals. As a result, there is a rising interest in novel antifungal agents, including a variety of synthetic and natural antimicrobial compounds [28]. Since the beginning of the 20th century, therapeutic choices have relied on synthesizing new drugs based on identified molecular pathways. Although the western medicine approach was declared effective in some situations, the reported lack of efficacy and serious adverse effects [29], have directed attention towards the medicinal capabilities of plant-derived products [15]. In this respect, the World Health Organization has proclaimed the importance of phytotherapy in primary health care, thereby encouraging further research to explore the potential of plants and their detailed mechanisms of action [30].

Pomegranate, a fruit of the *Punica granatum* L. (Lythraceae) tree, is native to the geographic area covering modern Iran and Iraq. Notably, the characteristics of this fruit, such as its color, flavor, and antioxidant properties, appear to be influenced by environmental factors [31]. Importantly, pomegranate, including its seeds, has been shown to possess significant antioxidant and anti-inflammatory properties [32,33], which are likely attributed to its high polyphenol content [34], particularly anthocyanins, 3-glucosides, delphinidin, cyanidin, pelargonidin [35], ellagitannins, ellagic acid, and other flavonoids [36]. The antimicrobial properties of pomegranate have recently garnered considerable attention due to their effect on multiple microbial species. In this regard, punicalagin, found in pomegranate peel, has been reported to exhibit potent antifungal activity against *C. albicans* and *Candida parasitosis* [37]. On the other hand, green tea has gained widespread attention due to its beneficial effects across multiple disease models [38]. More specifically, the *Camellia sinensis* (L.) Kuntze (Theaceae) species has demonstrated anti-cancer and anti-inflammatory properties [39]. Furthermore, tea polyphenols have shown antimicrobial potential against several human pathogens [40]. Given its potential antifungal activity, we investigated a standardized polyphenol-rich formulation derived from green tea and pomegranate. This product, commercially known as *Viroelixir*, is a blend of plant-derived polyphenols that may represent a promising in vitro antifungal candidate. In this study, our primary objective was to evaluate the antifungal potential of *Viroelixir* in vitro against the *C. albicans* model. Overall, we investigated its effect on fungal growth and mortality, sugar content, environmental pH, biofilm formation, pathogen phenotype, ROS production, and the expression of virulence determinants, including hydrolytic enzymes and adhesins. Additionally, we assessed its impact on fungal adhesion and host epithelial cells immunity and defense system.

## 2. Results

### 2.1. Viroelixir Inhibits C. albicans Growth and Interferes with Cell Viability

Based on component analysis previously reported [41], *Viroelixir* contains a defined mixture of bioactive polyphenols, including catechins such as EGCG from green tea and ellagitannins such as punicalagin and ellagic acid from pomegranate. These compounds are known to exert antimicrobial and anti-virulence effects and have been associated with antimicrobial activity in this study.

**Figure 1:**
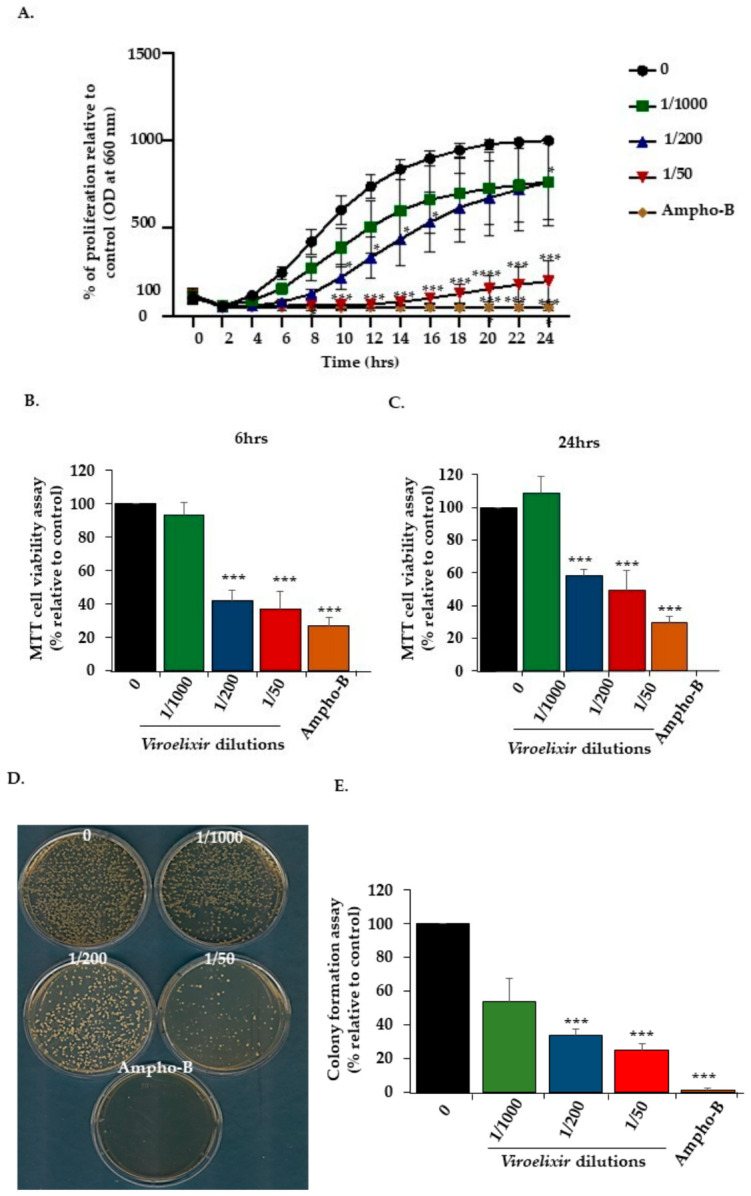
*Effect of Viroelixir on C. albicans growth and viability.* (**A**) Optical density recorded at 660 nm (*n* = 4 and quadruplet per experiment) over 24 h to reflect *C. albicans* growth. Comparison was performed relative to untreated *C. albicans* at the same time point. (**B**) MTT cell viability and proliferation assay (*n* = 4, quadruplicates per experiment) performed after 6 h of treatment with different *Viroelixir* dilutions (1/1000, 1/200, and 1/50). (**C**). MTT cell viability and proliferation assay (*n* = 4, quadruplicates per experiment) performed after 24 h of treatment with different *Viroelixir* dilutions (1/1000, 1/200, and 1/50). (**D**) Colony formation potential (*n* = 5) under *Viroelixir* effect. (**E**) Quantitative data corresponding to the number. The results are presented as a percentage, with 100% representing the number of colonies in untreated controls (*n* = 4). Antifungal drug *Ampho-B* (5 µg/mL) was used as the positive control. The Student’s *t*-test is a statistical test used to determine whether the difference between the control group and the *Viroelixir*-treated group is statistically significant. * *p* < 0.05, *** *p* < 0.001 and **** *p* < 0.0001 were considered statistically significant.

The effect of *Viroelixir* on *C. albicans* growth was evaluated by measuring the optical density (OD) variation at 660 nm every 2 h over a period of 24 h (Figure 1A) and confirmed by MTT assay at 6 h and 24 h (Figure 1B) and by colony formation on solid agar plates (Figure 1C,D). Exposure of *C. albicans* to *Viroelixir* resulted in concentration-dependent growth inhibition, with a minimum inhibitory concentration (MIC) corresponding to *Viroelixir* diluted at 1/50 of *v*/*v* of the stock solution. At 1/50, the effect of *Viroelixir* was comparable to that of the antifungal drug *Ampho-B* (5 µg/mL). The untreated *C. albicans* (0) increased from 100% to nearly 1000% after 14 h while *C. albicans* treated with the IC50 concentration (around of dilution 1/200) showed a 350% growth increase, reflecting a 70% suppression of proliferation. At a 1/50 (*v*/*v* of the stock solution) dilution of *Viroelixir*, a growth inhibition based on OD measurements. was observed, with untreated *C. albicans* (0) proliferating to 1000% after 24 h, while the treated *C. albicans* barely reached 100%, indicating significant inhibition (Figure 1A). These data were confirmed by the MTT assay (metabolic activity associated with viability, not direct viability) after 24 h of *Viroelixir* treatment, showing a significant decrease in viability, from 100% to 43% at the IC50 (1/200). The same pattern was observed after 6 h of treatment and *Ampho-B* (5 µg/mL) showed parallel efficacy to *Viroelixir* (Figure 1B). The extract fungicidal activity towards *C. albicans* was further confirmed by monitoring colony formation on solid agar plates. At this level, *Viroelixir* strongly prevented colony formation starting 1/1000 dilution (Figure 1C,D).

### 2.2. Effect of Viroelixir on Media pH and Carbohydrate Content

**Figure 2:**
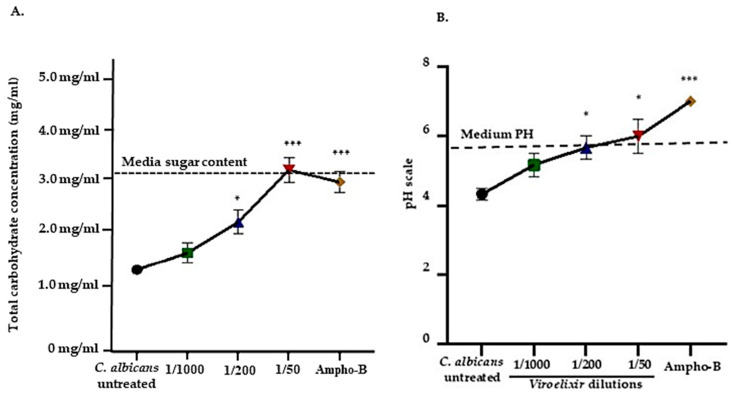
*Effect of Viroelixir on carbohydrate content and media pH.* (**A**) Total carbohydrate content (*n* = 3). (**B**) Evaluation of medium pH variation (*n* = 3) in culture media. The antifungal agent *Ampho-B* (5 µg/mL) served as the positive control, and comparisons are presented against untreated *C. albicans* cultures. Different colors represent the experimental conditions: untreated *C. albicans* (black), *Viroelixir* at 1/1000 (green), 1/200 (blue), 1/50 (red), and *Ampho-B* (orange) All data are expressed as mean values ± SEM. Student’s *t*-test is a statistical test used to test whether the difference between the control group and *Viroelixir* treated group is statistically significant or not. * *p* < 0.05, and *** *p* < 0.001 are considered as statistically significant.

Carbohydrate content in culture supernatants was measured using a colorimetric method to quantify sugars, as described in Section 4. As shown in Figure 2A, the higher residual glucose concentration in the media following *Viroelixir* and *Ampho-B* (5 µg/mL) treatment indicates reduced nutrient consumption due to fungal growth inhibition. Under our experimental conditions, glucose was initially provided at a final concentration of 1 mg/mL. The effect of *Viroelixir* on acid production by *C. albicans* was evaluated by monitoring changes in medium pH. Briefly, a decrease in pH was observed after 24 h of incubation with *C. albicans*, confirming acid production by the fungus. In contrast, a more neutral pH was recorded in cultures supplemented with *Viroelixir* and *Ampho-B* (5 µg/mL) compared to untreated controls. Although this shift may partly reflect reduced fungal viability, the increase in Sabouraud medium pH from 5.6 to approximately 6–7 in the presence of *Viroelixir* (1/50) and *Ampho-B* (5 µg/mL) may also suggest the production of basic metabolites. These findings indicate that antifungal treatments may alter *C. albicans* metabolic activity, particularly pathways involved in acid production (Figure 2B).

### 2.3. Viroelixir Counteracts Biofilm Formation

**Figure 3:**
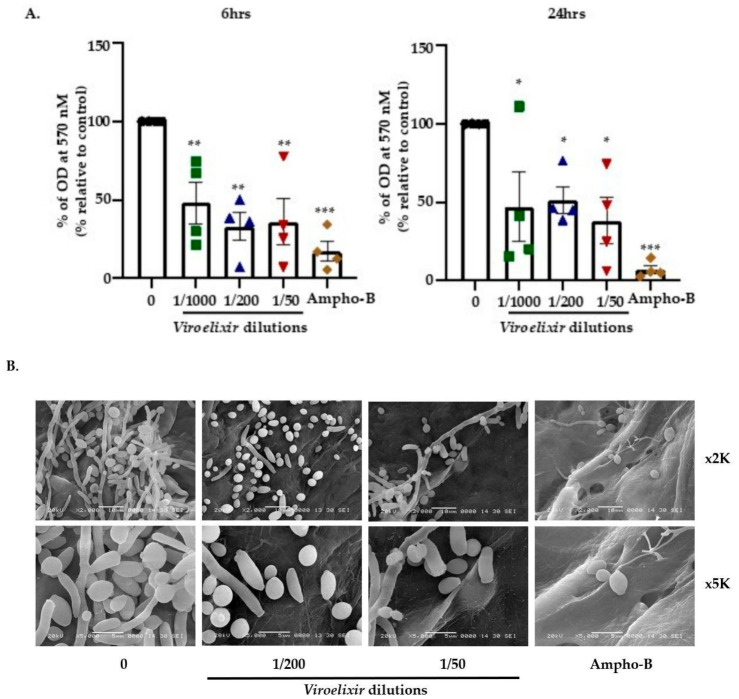
Effect of *Viroelixir* on biofilm formation. (**A**) Crystal Violet staining (*n* = 4) performed after treatment of fungal cells with different dilutions of *Viroelixir* (0, 1/1000, 1/200, 1/50 of *v*/*v* of the stock solution) for a total duration of 6 and 24 h. Antifungal agent *Ampho-B* (5 µg/mL) served as a positive control, and comparisons are presented against untreated controls. Different colors represent the experimental conditions: untreated control (black), *Viroelixir* at 1/1000 (green), 1/200 (blue), 1/50 (red), and *Ampho-B* (orange). All data are expressed as mean values ± SEM. Student’s *t*-test is a statistical test used to test whether the difference between the control group and *Viroelixir* treated group is statistically significant or not. * *p* < 0.05, ** *p* < 0.005, and *** *p* < 0.0005 are considered statistically significant. (**B**) Scanning electron microscopy images (*n* = 3) corresponding to 6 days of *C. albicans* biofilm evolution on porous membrane scaffolds following exposure to either *Viroelixir* or the positive control *Ampho-B* (5 µg/mL). Comparisons were performed relative to the untreated control.

Early biofilm formation, assessed at 6 and 24 h using the crystal violet assay, revealed that *Viroelixir*, reduced the biofilm formation after 6 h of *Viroelixir* exposure compared to untreated controls. Similar results were observed with crystal violet after 24 h of *Viroelixir* treatment (Figure 3A). As indicated in the Section 4, the biofilm formation assay was performed on a CollaTape membrane over 6 days, and the results were visualized by scanning electron microscopy (SEM) (Figure 3B). Overall, *Viroelixir* clearly inhibited biofilm formation starting at a 1/200 dilution. Interestingly, the images obtained with *Viroelixir* at 1/50 were comparable to those of the positive control, *Ampho-B* (5 µg/mL). Another important observation is that the non-virulent yeast phenotype was predominant following exposure to the natural product extract.

### 2.4. Viroelixir Favors the Elimination of C. albicans Virulent Forms

**Figure 4:**
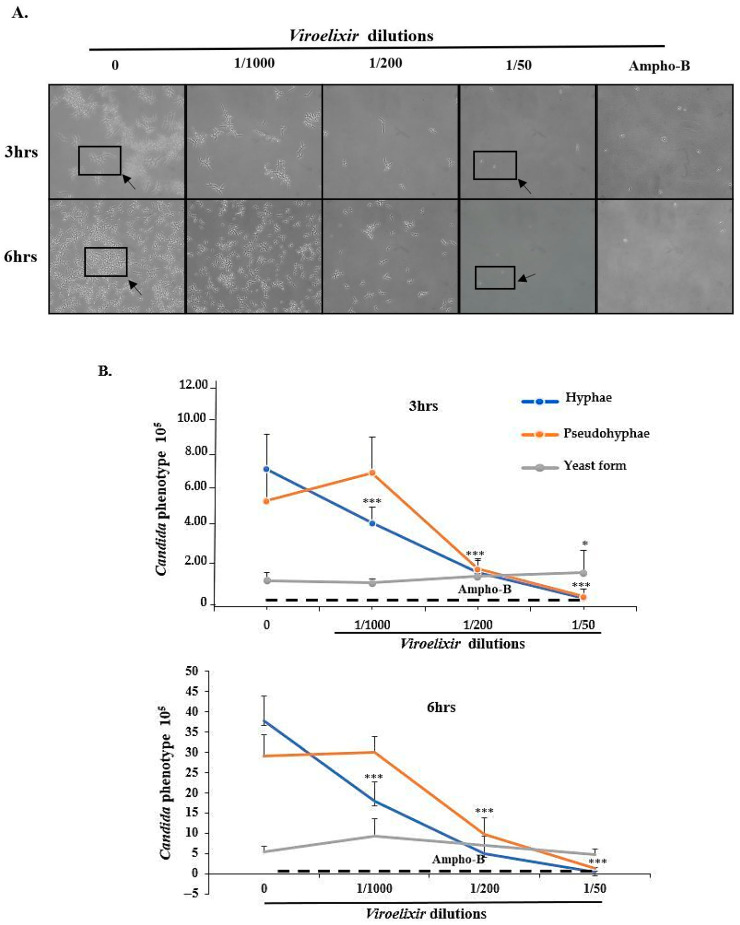
*Effect of Viroelixir on C. albicans morphology.* (**A**) *C. albicans* forms corresponding to yeast forms, pseudohyphae, and hyphal phenotypes were detected microscopically (*n* = 3). (**B**) These forms were counted (*n* = 3) after 3 and 6 h exposure to *Viroelixir* at 1/1000, 1/200, and 1/50 under hyphal growth conditions. *Ampho-B* (5 µg/mL) served as the positive control, and comparisons are presented against untreated controls. The dashed horizontal line represents the *Amphotericin B* (*Ampho-B*) treatment used as a reference control. All data are expressed as mean values ± SEM. * *p* < 0.05 and *** *p* < 0.001 were considered as statistically significant.

Due to its association with invasion and biofilm formation, *C. albicans* morphology was examined at 3 and 6 h of culture under hyphae-inducing conditions (10% FBS at 37 °C). Our data showed no effect on phenotypic switching. However, a decrease in hyphal formation was observed at a dilution of 1/1000 at both time points. A similar trend was observed for pseudohyphae, with a marked reduction at a dilution of 1/200. At this dilution, pseudohyphal formation was strongly reduced at 3 h. Although no significant difference was observed in yeast form numbers, this less virulent form gradually became dominant as the *Viroelixir* dilution increased from 1/1000 to 1/50. Interestingly, at the highest concentration tested (1/50), *Viroelixir* was almost as effective as the *Ampho-B* (5 µg/mL) positive control (Figure 4A,B).

### 2.5. Viroelixir Induces C. albicans Dead Cells “(PI-Positive)”, Possibly Through a ROS-Dependent Mechanism

**Figure 5:**
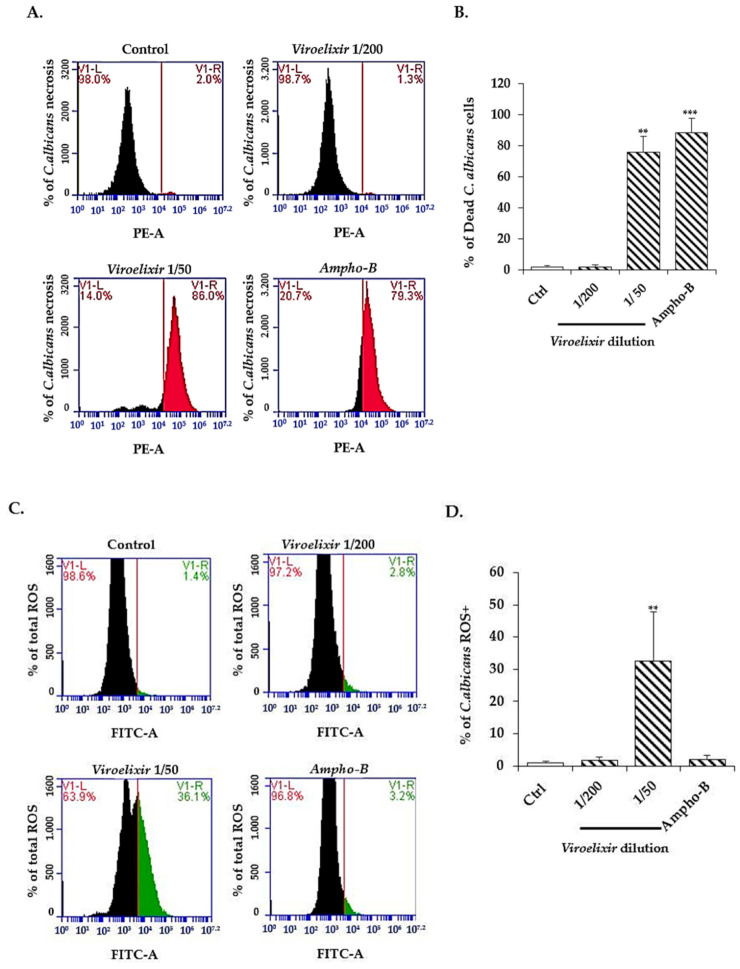
*Viroelixir controls C. albicans growth via necrosis/ROS pathways.* (**A**) Flow cytometry analysis of necrotic *C. albicans* using propidium iodide (PI) stain. The panels show positively marked *C. albicans* cells, under various treatment conditions (0, *Viroelixir* 1/200, *Viroelixir* 1/50 and *Ampho-B*) (5 µg/mL) In flow cytometry histograms, black peaks represent the total cell population, while colored regions indicate gated PI-positive cells. (**B**) Representative data presented as mean values ± SEM. (**C**) Flow cytometry panels showing total ROS production in *C. albicans* under the same conditions. Black histograms represent the total population, whereas colored regions indicate ROS-positive gated cells. (**D**) Summary of 4 independent experiments following statistical analysis. The Student’s *t*-test is a statistical test used to determine whether the difference between the control group and the *Viroelixir*-treated group is statistically significant. Significance is indicated as ** *p* < 0.01, and *** *p* < 0.001.

Following 24 h *Viroelixir* (1/200, and 1/50 of *v*/*v* of the stock solution) and *Ampho-B* treatment, we investigated *C. albicans* dead cells and oxidative stress. Flow cytometry analysis showed a significant shift towards *C. albicans* dead cells. More specifically, the percentage of dead cells increased from 1.95 ± 0.45% in untreated *C. albicans* cultures to 88.0 ± 2.96% and 90.7 ± 5.16% in the presence of 1/50 *Viroelixir* and the positive control *Ampho-B* (5 µg/mL), respectively (Figure 5A,B). Of note, a significant change in total ROS levels was observed only following exposure to *Viroelixir* 1/50. In particular, the percentage of ROS positive cells increased more than threefold under this condition, suggesting that additional or alternative mechanisms may contribute (Figure 5C,D).

### 2.6. Viroelixir Treatment Reduces the Transcript Levels of Virulence-Associated Genes

**Figure 6:**
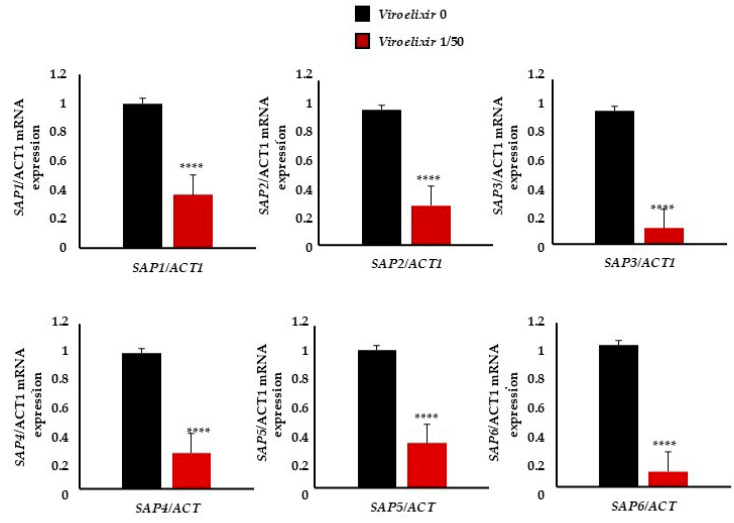

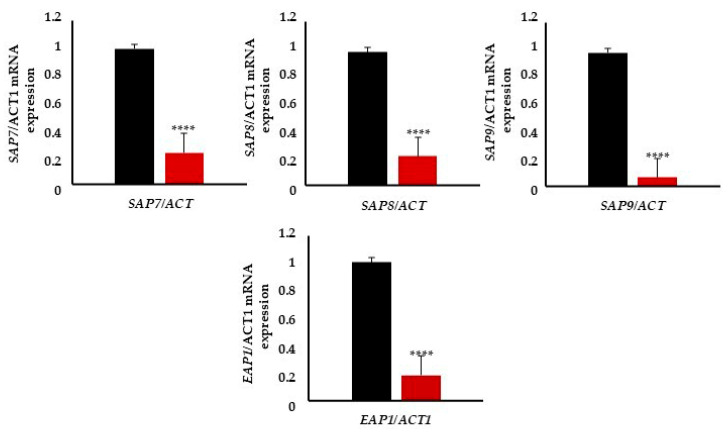
*Relative mRNA expression of SAP1–SAP9 and EAP1 normalized to ACT1*. Data are presented as mean ± SEM of independent experiments (*n* = 3). Statistical significance was determined using one-way ANOVA. **** *p* < 0.0001 compared to untreated control. Control (untreated) and *Viroelixir* (1/50 dilution).

To understand the anti-virulence mechanisms of action, we evaluated *Viroelixir* impact on EAP1 adhesion protein and SAPs hydrolytic enzymes expression. To ensure that differences in mRNA levels were not due to variations in cell number, samples were normalized prior to RNA extraction using an equivalent number of cells (based on CFU/OD measurements). These enzymes are known to play a key role in adhesion, tissue invasion, and immune evasion. Our results showed that treatment with *Viroelixir* at 1/50 dilution significantly reduced the mRNA levels of EAP1 and aspartyl proteinases family (SAP1 to SAP9) by more than 2.5-fold. *Viroelixir* treatment (1/50) significantly reduced the mRNA expression levels of EAP1 and the aspartyl proteinase family (SAP1–SAP9), with expression levels ranging from 0.36 ± 0.14 (SAP1), 0.28 ± 0.11 (SAP2), 0.15 ± 0.012 (SAP3), 0.25 ± 0.089 (SAP4), 0.32 ± 0.11 (SAP5), 0.11 ± 0.05 (SAP6), 0.23 ± 0.06 (SAP7), 0.21 ± 0.10 (SAP8), and 0.065 ± 0.02 (SAP9), as well as 0.18 ± 0.07 for EAP1, compared to untreated controls (set to 1) (Figure 6).

### 2.7. Viroelixir Causes an Irreversible Damage to C. albicans Adhesion Potential

**Figure 7:**
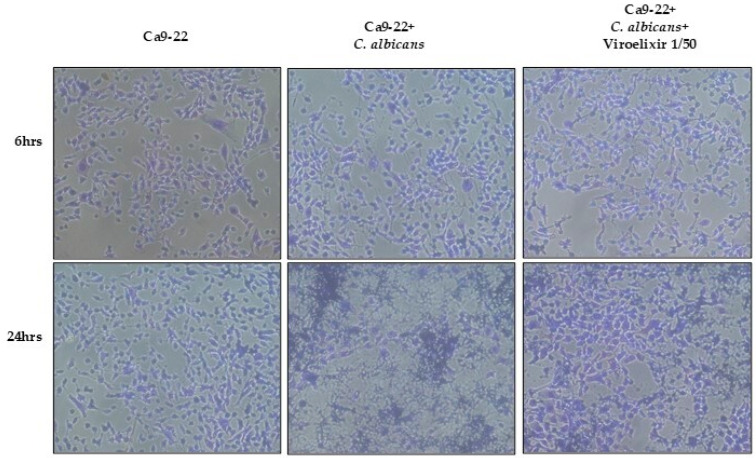
*Effect of Viroelixir on C. albicans adhesion potential. C. albicans* adhesion to gingival epithelial cells was assessed microscopically (*n* = 3) using the crystal violet staining assay. Overall, gingival epithelial cell monolayers were exposed for 6 and 24 h to *C. albicans* whether pre-treated or not with *Viroelixir* at 1/50.

*C. albicans*-gingival epithelial cells co-cultures were performed to confirm the irreversible antimycotic potential of *Viroelixir*, primarily at the level of adhesion. Briefly, when *C. albicans* were pretreated with the highest concentration tested of *Viroelixir* at 1/50 for 24 h. Following the 24 h pre-treatment with *Viroelixir*, *C. albicans* cells were collected, quantified, and re-adjusted to the same cell density prior to co-culture with epithelial cells. This normalization step ensured that an equivalent number of fungal cells was used across all conditions, thereby allowing a direct evaluation of adhesion capacity independent of cell viability the adhesion capacity of the fungal cells was significantly reduced. This is evident after 24 h co-culture period where pre-treated *C*. *albicans* cells were not capable of adhering to the cell monolayer in a similar manner to untreated *C. albicans*. Consequently, the cytotoxicity and morphological changes in gingival epithelial cells caused by the virulent *C. albicans* form were almost completely absent, leaving the epithelial cells in a state comparable to uninfected cells. The few *C. albicans* cells that did adhere to gingival cells after *Viroelixir* pre-treatment exhibited a less virulent phenotype, primarily known as yeast forms (Figure 7). The results are presented as qualitative observations only. Importantly, under our experimental conditions, a clear and reproducible reduction in adherent *C. albicans* cells was consistently observed in the *Viroelixir*-treated group compared to untreated controls, supporting the reported trend.

### 2.8. Viroelixir Attenuates Epithelial Cytokine and Antimicrobial Responses

**Figure 8:**
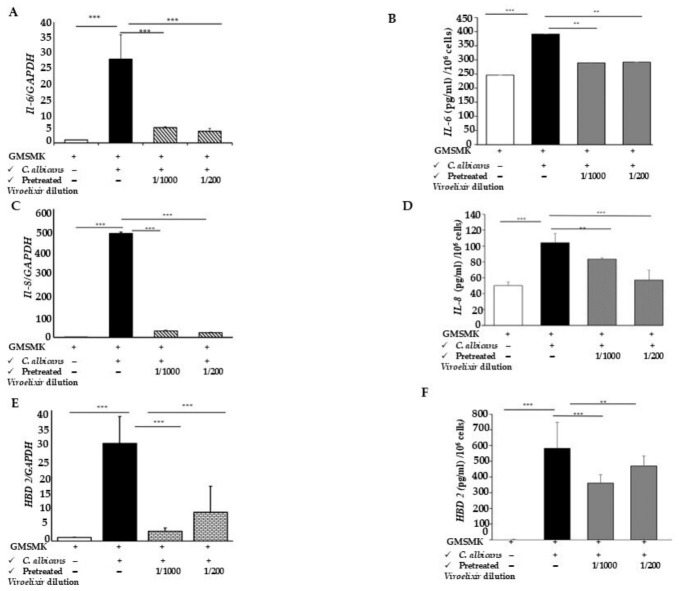
*Viroelixir attenuates C. albicans virulence and relieves GMSMK defense mechanisms.* (**A**) IL-6, (**C**) IL-8, and (**E**) hBD-2 mRNA expression levels in GMSMK cells following co-culture with *C. albicans*, whether pre-exposed or not to *Viroelixir* at 1/1000 and 1/200. mRNA expression was analyzed by qRT-PCR and normalized to GAPDH. (**B**) IL-6, (**D**). IL-8, and (**F**). hBD-2 protein levels in culture supernatants quantified by ELISA, in the bar charts, white bars represent GMSMK cells alone, black bars represent GMSMK cells in contact with untreated *C. albicans*, and gray bars represent GMSMK cells in contact with *Viroelixir*-treated *C. albicans* at the indicated dilutions. Data are presented as mean ± SEM. ** *p* < 0.01, and *** *p* < 0.001 are considered as statistically significant.

In this section, we examined the effect of *Viroelixir* pretreatment, i.e., *C. albicans* virulence attenuation, on epithelial cell defense mechanisms. To this end, *C. albicans* cells (pretreated or not with *Viroelixir*) were co-cultured with non-oncogenic human epithelial cell lines (GMSMK). To ensure that differences in mRNA levels were not due to variations in cell number, samples were normalized prior to RNA extraction using an equivalent number of cells (based on CFU/OD measurements). The ability of epithelial cells to express and secrete pro-inflammatory cytokines (such as IL-6 and IL-8), as well as antimicrobial peptides (e.g., hBD-2), was assessed by real-time PCR and ELISA. As shown in Figure 8, virulent *C. albicans* significantly induced IL-6, IL-8, and hBD-2 mRNA expression and protein secretion in GMSMK cells. In contrast, *Viroelixir* pretreatment reduced *C. albicans* virulence and adhesion potential, thereby attenuating the activation of epithelial cell defense responses. This was reflected by overall lower mRNA expression and secretion levels of pro-inflammatory cytokines (IL-6 and IL-8) and the antimicrobial peptide hBD-2 (Figure 8). Similar trends were observed for IL-1β, hBD-1, hBD-3, and hBD-4 (Figures S1–S4).

### 2.9. Pretreatment by Viroelixir Inhibits Signaling Pathways Induced by C. albicans Infection in the Oral Cavity

**Figure 9:**
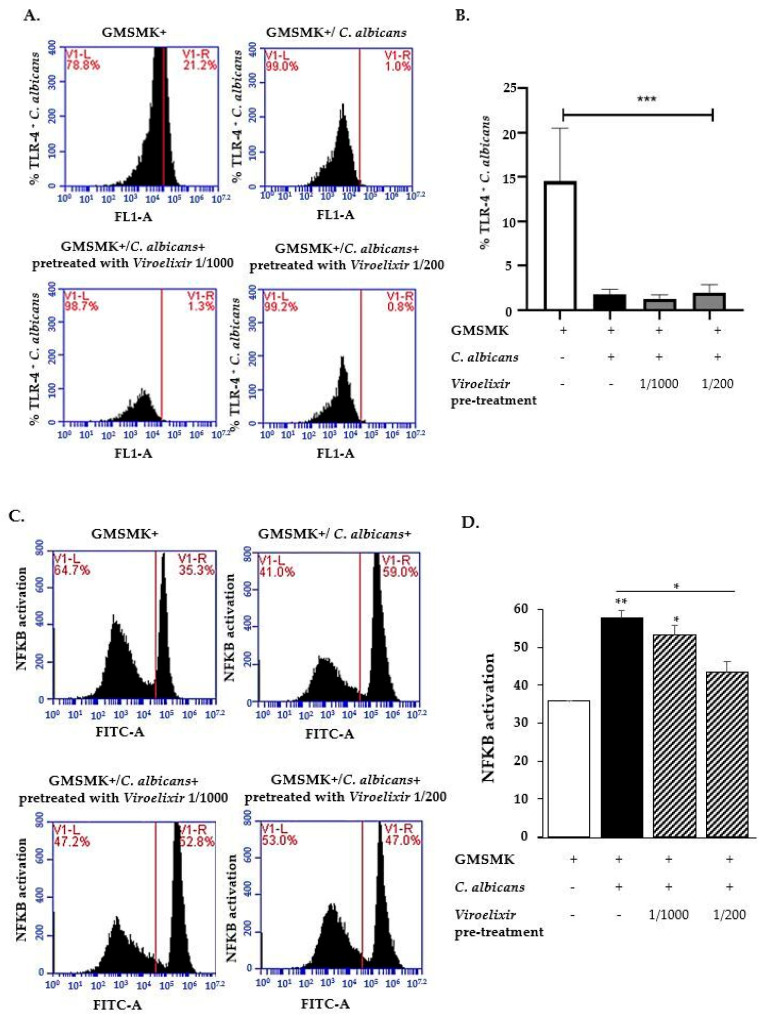
*Viroelixir* modulates *C. albicans* infection via the TLR4-NF-κB signaling pathway. (**A**) Flow cytometry analysis of TLR4 expression in *C. albicans*-infected GMSMK cells and *C. albicans* pretreated with different dilutions of *Viroelixir* (1/1000 and 1/200) before infection of GMSMK cells. The panels show GMSMK cells, marked for TLR4 positivity, following infection with untreated *C. albicans* and *C. albicans* pretreated with various dilutions of *Viroelixir* (1/1000 and 1/200). In the bar charts, white bars represent GMSMK cells alone, black bars represent GMSMK cells in contact with untreated *C. albicans*, and gray bars represent GMSMK cells in contact with *Viroelixir*-treated *C. albicans* at the indicated dilutions (**B**) Summary of three independent experiments. (**C**) Flow cytometry analysis of NF-κB activation in *C. albicans*-infected GMSMK cells and *C. albicans* pretreated with different dilutions of *Viroelixir* (1/1000 and 1/200) before infection of GMSMK cells. The panels show GMSMK cells, marked for p65 positivity, following infection with untreated *C. albicans* and *C. albicans* pretreated with various dilutions of *Viroelixir* (1/1000 and 1/200). (**D**) Summary of three independent experiments. The analysis data are presented as mean values ± SEM. Statistical significance is indicated as * *p* < 0.05, ** *p* < 0.05, and *** *p* < 0.005 are considered as statistically significant.

As shown in Figure 9A,B, TLR4 expression tended to decrease in GMSMK cells infected with *C. albicans*, regardless of whether the fungus was treated with *Viroelixir* or not, compared to non-infected cells. To ensure that differences in mRNA levels were not due to variations in cell number, samples were normalized prior to RNA extraction using an equivalent number of cells (based on CFU/OD measurements). NF-κB activation was significantly increased in GMSMK cells following infection with untreated *C. albicans* (57.83 ± 1.85%) compared to non-infected cells (34.8 ± 0.16%) (Figure 9C,D). Pre-treatment of *C. albicans* with *Viroelixir* reduced NF-κB activation in a dose-dependent manner. Specifically, infection with *C. albicans* pre-treated at 1/1000 resulted in an activation level of 53.4 ± 2.44%, while pre-treatment at 1/200 further reduced activation to 43.46 ± 2.90%.

These reductions were statistically significant compared to cells infected with untreated *C. albicans* (*p* < 0.05), with a more pronounced decrease observed at the 1/200 dilution. Data represent the mean ± SEM of three independent experiments.

## 3. Discussion

The antifungal effects observed in this study are consistent with the known antimicrobial activity of catechins and ellagitannins identified in the *Viroelixir* formulation, including EGCG and punicalagin, as confirmed in our previous component analysis study.

Natural antifungal compounds derived from plant sources have been increasingly investigated as potential alternatives or complements to conventional antifungal therapies. These compounds are generally characterized by multi-target activity, relatively low toxicity, and the ability to modulate microbial virulence [42]. The present study illustrates the impact of *Viroelixir*, a novel pomegranate and green tea extract, on the growth and virulence determinants of *C. albicans*. Although both plants exhibit antimycotic potential, the introduction of the *Viroelixir* mixture is expected to provide greater benefits due to the presence of multiple active ingredients targeting diverse molecular pathways. This multi-targeted approach not only helps limit or delay the development of resistance but also exerts anti-inflammatory effects, enhancing its overall efficacy [42]. According to the literature, green tea has been demonstrated to be effective against several microbial species and tea catechins have been shown to act synergistically with *Ampho-B* and *fluconazole* against both susceptible and resistant *C. albicans* strains [43]. Overall, the protective effect of green tea polyphenols includes *C. albicans* biofilm formation and exopolysaccharide secretion. The active ingredients in tea are also known to influence the expression of surface proteins implicated in adhesion, such as hyphal wall protein 1 (HWP1) and agglutinin-like sequence 3 (ALS3) [44]. On the other hand, the antimicrobial potential of pomegranate products has been validated against a wide range of pathogenic bacterial species [45,46,47] as well as certain fungi [48]. take. In this regard, one study demonstrated that both crude pomegranate extract and its pure compound, punicalagin, exhibit antifungal effects against *C. albicans*, with their activity further enhanced by the supplementation of *fluconazole*, a well-known drug for the treatment of candidiasis [37], which is consistent with our findings.

After conducting the first series of experiments, we were able to confirm that *Viroelixir* was associated with reduced fungal growth and increased ROS levels. Based on previous studies with flavonoid-rich compounds, this increase in oxidative stress may contribute to fungal cell damage [49]. However, the precise mechanisms underlying this effect remain to be fully elucidated.

This mechanism could play a key role in the antifungal effects of *Viroelixir*, further corroborating its potential as an effective treatment against *C. albicans* infections. Although sugar sensing and carbon source utilization are generally considered key factors for more virulent behavior such as increased adhesion, transition to hyphae, biofilm formation, matrix polysaccharide secretion, hydrolytic enzyme activity, cell wall structural organization, oxidative stress resistance, invasion, and antifungal drug tolerance [50,51], the increase in carbohydrate levels observed after administering *Viroelixir* does not seem to potentiate virulence. This is most likely a direct result of fungal elimination, leading to reduced consumption of carbon sources. It is important to note that fungal species can adapt and proliferate under both nutrient-rich and nutrient-poor conditions, reflecting real-life situations where different glucose concentrations are available at various sites of *C. albicans* infection [51].

Having demonstrated *Viroelixir*’s impact on growth inhibition, we aimed to investigate its effect on biofilm formation, given its strong association with pathogenicity. It is important to highlight that fungal biofilm do not only protect *C. albicans* from host immune defenses but are also closely linked to the development of drug resistance. The increase in resistance and the need for higher drug dosages may be attributed to several factors, including a lack of effect on adhesion, enhanced drug metabolism, and drug sequestration and diffusion prevention by exopolysaccharides. In this context, *Viroelixir*’s anti-biofilm properties were evident even at higher dilutions. Based on evidence showing that biofilm initiation and *C*. *albicans* virulence is promoted by its morphological transition from yeast formto hyphal form; yeast morphological changes were also investigated. It is not surprising that hyphal assembly marks biofilm formation. At this level, the ability of hyphae to act as a supporting scaffold by adhering to one another and to other cell morphologies in polymicrobial biofilms is critical for maintaining architectural stability [52,53]. Hyphae also facilitate host tissue invasion through penetration and induce endocytosis. Among its various properties, this pathogenic phenotype expresses morphology-dependent adhesins, invasins, proteases, and secretes specific toxins, such as candidalysin, which is crucial for damaging host cells and contributes to the development of systemic candidiasis and mortality. The morphological switch is essential for virulence, as different *C. albicans* forms affect immune recognition differently. Hyphae stimulate lower cytokine responses and alter the availability of surface immunostimulatory ligands [54,55,56]. The observed reduction in hyphal formation may be associated with decreased biofilm development and invasive potential, although this relationship requires further validation in more complex biological models.

Considering these findings, investigating the impact on the adhesion step was of utmost importance, as co-culture data confirmed the inhibition of *C. albicans* attachment to epithelial cells. In this respect, *Viroelixir* treatment was associated with the downregulation of several virulence-related genes, including EAP1 and members of the SAP family, suggesting a potential impact on adhesion and pathogenicity mechanisms [57]. Although SAPs are recognized as key virulence determinants due to their role in tissue degradation and phenotypic switching, their specific involvement in the attachment process remains not fully understood. Researchers suggest that surface SAPs might function as non-enzymatic adhesins, while secreted SAPs could alter host cell surface proteins to expose proper binding ligands [58]. Importantly, SAP1 to SAP7 genes were found to be present in 31 susceptible or resistant *C. albicans* isolates [58,59], highlighting the potential advantage offered by *Viroelixir* in managing fungal infections. It seems that the effect of our extract on SAPs can be further enhanced by favoring a more neutral pH. Although *C. albicans* can adapt to a broad range of pH fluctuations [60], it has been shown that proteolytic activity in *C. albicans* isolates from 20 clinical cases across five different infection sites, including the oral cavity, was detected at a more acidic pH [61]. In another study, the optimal pH for SAP activity was found to be 2.5–4 for SAP2, 3, and 8, while the remaining SAPs were most active at pH 5–6.5 [62]. Another important observation is that SAPs are linked to severe early childhood caries [63]. This is likely due to *C. albicans*’s ability to secrete organic acids [64], which aligns with our finding that *C. albicans* cells can acidify the culture media. Despite reports indicating a complete adverse effect on environmental pH due to amino acid metabolism contributing to ammonia release, the possible role of *Viroelixir* in the management of caries cannot be neglected as *C. albicans* behavior is susceptible to change based on carbon source availability (amino acids/glucose) [65].

In the current study, *Viroelixir* influenced *C. albicans* interaction with gingival epithelial cells, supporting its role in pathogen adhesion and host-mediated defense mechanisms. During oral infections, hyphae invade by penetrating epithelial cells, which responds by producing pro-inflammatory cytokines and triggering antimicrobial peptides, particularly those of the β-defensin family (hBDs) [66]. Notably, antimicrobial peptides such as hBD-2 and hBD-3 have been shown to reduce the invasive capacity of *C. albicans* and to modulate host defense pathways [67]. hBDs are also essential for controlling early mucosal infections and play a critical role in the induction of innate inflammatory mediators [68,69,70,71]. Persistent infection disrupts this balance, playing a key role in the pathogenesis of various inflammatory diseases [72]. Therefore, the pro-inflammatory cytokines (IL-1β, IL-6, and IL-8) and hBDs were analyzed in oral epithelial cells following infection with *C. albicans*. While *C. albicans* infection induced increased expression of pro-inflammatory cytokines and hBDs, less virulent forms, pretreated with *Viroelixir*, were shown to alleviate this defense response. This finding is similar to the effects observed in *C. albicans* under monolaurin treatment [73]. However, conflicting evidence exists regarding the extent to which modulation of fungal virulence directly translates into reduced host inflammatory responses, suggesting that additional host-dependent factors may be involved.

Evidence highlights the critical role of TLR4-mediated signaling, primarily through NF-κB, in protecting epithelial cells from *C. albicans* infection [74,75]. Receptor activation may also induce AP-1, while virulent *C. albicans* (hyphal forms) can activate the MAPK1 and FOS pathways, facilitating tissue invasion [76]. Hyphae invade by penetrating epithelial cells, which produce cytokines via the MAPK1 pathway, and trigger β-defensins with anti-*C. albicans* activity. Compared to virulent *C. albicans*, inflammatory cytokines and antimicrobial peptides were reduced to baseline levels. Interestingly, TLR4 recognizes both virulent and non-virulent strains, with varying immune responses depending on pathogen recognition. It also recognizes heat-killed *C. albicans* due to conserved mannans [77]. In our study, we observed a decrease in surface expression of TLR4 in epithelial cells infected with *C. albicans*, whether treated with *Viroelixir* or not, compared to non-infected cells. This apparent reduction may be explained by TLR4 internalization, a well-documented mechanism that occurs upon immune stimulation [78]. After recognizing fungal components, TLR4 is internalized from the plasma membrane into intracellular compartments such as Rab5^+^ early endosomes and Rab7^+^ late endosomes, where it continues to activate signaling pathways, including the TRIF-dependent cascade leading to NF-κB activation [79,80]. Upon recognition of pathogen-associated molecular patterns (PAMPs), TLR4 is internalized into Rab5-positive early endosomes, where it interacts with the adaptor protein TRAM and activates the TRIF-dependent signaling cascade. This pathway leads to the production of type I interferons and the expression of IFN-induced genes, as well as sustained activation of NF-κB [81]. Our findings are consistent with this mechanism since NF-κB activation remained significant despite the observed reduction in membrane-associated TLR4. These observations are consistent with previously described mechanisms of TLR4 internalization and intracellular signaling. The maintained NF-κB activation despite reduced surface TLR4 expression may reflect ongoing signaling from internalized receptors; however, additional investigations are required to confirm this hypothesis. In addition, differences in experimental conditions, including fungal strains and in vitro models, may influence the observed effects and should be considered when interpreting these results.

This study has several limitations that should be acknowledged. In addition, pH measurements were performed using indicator strips, which provide semi-quantitative estimates and may lack precision compared to electronic pH meters. This limitation may affect the accuracy of pH-related interpretations. First, the experiments were conducted using in vitro models, which do not fully recapitulate the complexity of the oral mucosal environment. Second, although significant associations were observed between *Viroelixir* treatment and modulation of fungal virulence and host responses, the underlying molecular mechanisms were not directly investigated. Third, the use of specific cell lines may limit the generalizability of the findings to other epithelial models. Additionally, only selected signaling pathways were evaluated, and other relevant pathways may also be involved. Finally, further studies using advanced models, such as engineered human oral mucosa or in vivo systems, will be necessary to confirm the translational relevance of these findings.

## 4. Materials and Methods

### 4.1. C. albicans Strain and Epithelial Cell Culture

*C. albicans*, a major opportunistic pathogen associated with candidiasis, was used in this study (reference strain ATCC MYA-2876; ATCC, Manassas, VA, USA). *C. albicans* was grown in Sabouraud dextrose broth (Becton Dickinson, Hong Kong, China; cat. #238230) containing 5 g/L peptone, 5 g/L peptic digest of animal tissue, 2 g/L pancreatic digest, and 20 g/L dextrose. The Sabouraud liquid medium was supplemented with glucose to a final concentration of 1 mg/mL and adjusted to pH 5.6. Glucose was used as the primary carbon source to support fungal growth and metabolic activity, which are essential for biofilm formation and virulence-related processes. The human oral squamous cell carcinoma cell line Ca9-22 (RIKEN BioResource Research Center, CVCL_1102, Ibaraki, Japan) and gingival epithelial cells GMSMK (provided by Dr. Daniel Grenier, Faculty of Dentistry, Laval University, Québec City, QC, Canada) were used in this study. Ca9-22 cells were cultured in RPMI 1640 medium (Gibco, Burlington, ON, Canada; cat. #10-040-CV) supplemented with 5% fetal bovine serum (FBS) (Thermo Fisher Scientific, Burlington, ON, Canada; cat. #10099-141), 0.2% *Amphotericin B* (*Ampho-B*) (Sigma-Aldrich, Oakville, ON, Canada; cat. #A2942), and 0.2% penicillin–streptomycin (P/S) (Sigma-Aldrich, St. Louis, MO, USA; cat. #P4333). GMSMK cells were maintained in DMEM medium (Gibco, Burlington, ON, Canada; cat. #11320033) supplemented with 10% FBS, 0.2% *Ampho-B*, and 0.2% P/S. Epithelial cells were cultured at 37 °C in a humidified atmosphere containing 5% CO_2_, whereas *C. albicans* cultures were incubated under standard aerobic conditions.

### 4.2. Viroelixir Agent Preparation

The *Viroelixir* agent, a standardized polyphenol blend derived from green tea (*Camellia sinensis* (L.) Kuntze, Theaceae) and pomegranate (*Punica granatum* L., Lythraceae), was provided by Dr. Gin Wu (Comcast, Taiwan, China). The extract was prepared by mixing green tea leaves and pomegranate peel powder (5 g each) in 100 mL of sterile water, followed by centrifugation and sterile filtration, as previously described. The preparation consisted of an aqueous extract obtained from a 1:1 mixture of green tea leaves and pomegranate peel powder. *Viroelixir* was supplied by the collaborator as a filtered liquid preparation (1 L batch). Upon receipt, the solution was aliquoted into sterile 5 mL tubes and stored at 4 °C until use. No specific batch number was assigned to this preparation. Component analysis performed in our previous study identified major bioactive constituents, including catechins (epigallocatechin gallate [EGCG] and epicatechin gallate), ellagitannins (punicalagin), ellagic acid, as well as other flavonoids and phenolic acids. The composition of the extract was previously characterized to confirm the presence of these major polyphenolic compounds, which are known for their antimicrobial, antioxidant, and anti-virulence properties. The stock solution (5% *m*/*v*) was stored at 4 °C until use.

### 4.3. Antifungal Agent Preparation

*Amphotericin B* (*Ampho-B*; Sigma-Aldrich, Oakville, ON, Canada; cat. #A2942) was used as a positive control at a final concentration of 5 µg/mL. The stock solution was prepared at a concentration of 250 µg/mL and sterile-filtered through a 0.22 µm pore-size membrane prior to use [82]. For all experiments, a working concentration of 5 µg/mL was used.

### 4.4. C. albicans Growth Assay

As detailed in our previous findings [83], *C. albicans* were seeded at 2 × 10^4^ cells per well of a 96-well plate and exposed to different dilutions of *Viroelixir* (1/1000, 1/200, and 1/50 of *v*/*v* of the stock solution). *Ampho-B* (5 µg/mL) was used as a positive control. *C. albicans* growth was assessed by reading the optical density at 660 nm over a total period of 24 h. Optical density values were automatically recorded every 2 h using an Agilent BioTek Synergy H1 microplate reader (Agilent BioTek Instruments, Winooski, VT, USA). This method ensures a quantitative and reproducible assessment of the effect of *Viroelixir* on *C. albicans* growth [84].

### 4.5. MTT Viability and Proliferation Assay

Cultures of *C. albicans*, initially at a density of 10^5^ cells/mL, were incubated for 6 and 24 h in the presence of *Viroelixir* (1/1000, 1/200, and 1/50 of *v*/*v* of the stock solution). Cell viability and proliferation were then assessed by measuring mitochondrial activity using the 3-(4,5-dimethylthiazol-2-yl)-2,5-diphenyltetrazolium bromide (MTT) assay. For further details, MTT (Millipore, Burlington, MA, USA; Cat# C501/CT02) was added to the Sabouraud medium containing the cells at a concentration of 0.5 mg/mL and incubated for 3 at 37 °C in the dark. Subsequently, the formazan crystals formed were dissolved in isopropanol containing 0.04% hydrochloric acid, and absorbance was measured at 550 nm using a Bio-Rad reader, as previously reported [82,85]. The results are represented as percentages using the following formula: % of proliferation = (treated cells OD/untreated cells OD) × 100. Four technical replicates and four to five biological replicates were carried out.

### 4.6. Effect of Viroelixir on Colony Formation

*C. albicans* cultures containing 10^3^ cells/mL were prepared in Sabouraud liquid medium. 20 µL of each condition were plated onto 60 mm diameter agar plates. Colonies were incubated at 37 °C for 24 h before being photographed and counted. The results are represented as percentages, with 100% corresponding to the number of colonies in untreated conditions [68]. Five biological replicates were carried out.

### 4.7. Effect of Viroelixir on Carbohydrate Content

Carbohydrate content was measured using the colorimetric method described by Dubois et al. [86]. Total sugar was quantified in the culture supernatants collected from *C. albicans* cultures after incubation under the experimental conditions. Briefly, 200 µL of supernatant was mixed with 100 µL of 5% phenol and 500 µL of H_2_SO_4_. The tubes were then incubated for 20 min at 30 °C, then cooled with water at 20 °C. Under the action of concentrated sulfuric acid, the hexoses and pentoses in the medium undergo dehydration to form furfural and 5-hydroxymethylfurfural derivatives, which react with phenol to form a yellowish complex. Optical density was measured at 485 nm using the Bio-Rad xMark microplate spectrophotometer (xMark^TM^ Microplate Absorbance Spectrophotometer (Bio-Rad Laboratories, Hercules, CA, USA). Carbohydrate concentrations were determined using a glucose standard curve and expressed as glucose equivalents (mg/mL). Three technical replicates and three biological replicates were carried out.

### 4.8. Measurement of Environmental pH

To evaluate the effect of *Viroelixir* on environmental pH, *C. albicans* biofilms were formed in the presence of various dilutions of this natural product (1/1000, 1/200, and 1/50 of *v*/*v* of the stock solution). Given that the pH of *Viroelixir* is 2.45, while that of *Ampho-B* (5 µg/mL) is 5.43, the overall change in the pH of Sabouraud culture medium (pH = 5.6) was recorded after 24 h. The pH was measured using pH indicator strips (MColorpHast^TM^ pH-indicator strips, universal indicator (pH 0–14), Millipore, Burlington, MA, USA; Cat# 1.09535.0001). Three technical replicates and three biological replicates were performed.

### 4.9. Crystal Violet Assay

It should be noted that biomass quantification reflects total attached biomass and does not directly correspond to viable cell counts (CFU). In addition, *C. albicans* at a concentration of 10^6^ cells/mL were exposed to different dilutions of *Viroelixir* for 6 and 24 h. The staining procedure involved discarding the culture medium, fixing the cells with 200 µL of cold methanol for 10 min, rinsing with PBS, and staining with a 1% crystal violet solution (Sigma-Aldrich, 548-62-9). The stained cells were then washed thoroughly with PBS to remove any excess dye. Afterward, the samples were left to air dry for 24 h before adding 400 µL of pure glacial acetic acid to dissolve the stain. Absorbance readings were recorded at 570 nm using a Bio-Rad xMark microplate spectrophotometer (Bio-Rad Laboratories, Hercules, CA, USA). All experiments were performed in at least three independents technical replicates and four biological replicates were carried out.

### 4.10. Biofilm Formation and Scanning Electron Microscopy

As per our previous study [85], *C. albicans* cells were seeded on a porous collagen scaffold to promote biofilm formation while maintaining structural integrity. Briefly, 5 mm × 5 mm samples of porous collagen scaffold (CollaTape^®^, Zimmer Dental Inc., Carlsbad, CA, USA) were placed in 24-well plates. The scaffolds were rinsed twice with culture medium and inoculated with *C. albicans* (10^5^ cells), followed by incubation for 60 min at 30 °C without shaking to allow initial adhesion.

Fresh Sabouraud medium was then added in the presence or absence of different dilutions of *Viroelixir* (0, 1/200, and 1/50). Two control conditions were included: untreated *C. albicans* as a negative control and cells treated with *Ampho-B* (5 µg/mL) as a positive control.

The *C. albicans*-seeded scaffolds were incubated at 30 °C for 6 days to allow biofilm development. Incubation at 30 °C was selected to promote optimal biofilm formation and maturation, as commonly described in in vitro models. The culture medium containing *Viroelixir,* or *Ampho-B* was renewed every 48 h to maintain consistent treatment conditions.

After the 6-day incubation period, biofilms were fixed with 4% (*v*/*v*) paraformaldehyde overnight at room temperature. Samples were then washed with phosphate-buffered saline (PBS) and processed for scanning electron microscopy (SEM) analysis by a specialized imaging facility using standard preparation procedures, including dehydration, drying, and sputter-coating before imaging.

All experiments were performed in at least three independent experiments, each conducted in triplicate.

### 4.11. Effect of Viroelixir on C. albicans Transition

*C. albicans* were grown in 6-well plates at 10^6^ cells per mL and exposed to varying dilutions of *Viroelixir*. Negative controls consisted of *C. albicans* cultures without Viroelixir, while positive controls corresponded to *Ampho-B* (5 µg/mL) supplementation. 10% FBS (Thermo Fisher, Burlington, ON, Canada, cat #10 099-141) was added, and the cells were incubated at 37 °C for 3 and 6 “h” to induce phenotypic transition from yeast to hyphal form. At the end of each incubation period, images were captured using a Nikon Eclipse TS100 microscope at 20× magnification. Trypan blue was used to facilitate the counting of *C. albicans* phenotypes (hyphae, pseudohyphae, and yeast forms). All experiments were performed in at least three independent experiments, each conducted in triplicate.

### 4.12. Cell Viability Analysis by Propidium Iodide (PI) Staining

Cell death was evaluated using Propidium Iodide (PI) stainingKit (BioLegend, San Diego, CA, USA). Briefly, after 24 h of treatment with Viroelixir (1/200 and 1/50 dilutions) or *Ampho-B* (5 µg/mL) (positive control), *C. albicans* cells (10^7^ cells/mL) were centrifuged at 500× *g* for 5 min and washed with sterile PBS. The cell pellets were then stained with 10 µL PI after being resuspended in 100 µL PBS. Following 15 min incubation at room temperature in the dark, 400 µL of assay buffer was added to each tube, and flow cytometry analysis was performed using the BD Accuri C6 Plus Flow Cytometer (BD Biosciences, San Jose, CA, USA). A total of 50,000 events per sample were collected and classified as necrotic cells (PI+). All experiments were performed in at least three independent experiments, each conducted in triplicate.

### 4.13. Reactive Oxygen Species (ROS) Analysis by Flow Cytometry

Oxidative stress in *C. albicans* was assessed by flow cytometry using a total ROS detection kit (ImmunoChemistry Technologies, Burlington, ON, Canada), according to the manufacturer’s instructions. Briefly, after 24 h of treatment with *Viroelixir* (1/200 and 1/50 dilutions of *v*/*v* of the stock solution) or *Ampho-B* (5 µg/mL) (positive control), *C. albicans* cells (10^7^ cells/mL) were centrifuged at 500× *g* for 5 min and washed with sterile PBS. The cells were then labeled with 10 µL ROS detection reagent (previously reconstituted in DMSO and diluted in assay buffer) after being resuspended in a total volume of 490 µL assay buffer. After incubation in the dark at 30 °C for 1 h, ROS levels were measured using a green fluorescent ROS-sensitive probe (excitation/emission ~488 nm) and analyzed using a BD Accuri^TM^ C6 Plus Flow Cytometer (BD Biosciences, San Jose, CA, USA). All experiments were performed in at least three independent experiments, each conducted in triplicate.

### 4.14. Reverse Transcription and Polymerase Chain Reaction

*C. albicans* (10^7^ cells) were either stimulated or not with 1/50 of *v*/*v* of the stock solution of *Viroelixir* for 24 h. Briefly, the samples were centrifuged for 10 min at 15,000 RPM. After centrifugation, 350 µL of Qiagen lysis buffer (Qiagen, Hilden, Germany, cat # 160035113) supplemented with 7 µL of ß-mercaptoethanol (Sigma-Aldrich, St. Louis, MO, USA, cat # 1003664856) was added to the pellets, along with 200 µL of magnetic beads. To promote membrane lysis, the samples were vigorously vortexed and exposed to a Biospec Products mini bead beater machine (Biospec Products, Bartlesville, OK, USA). Total RNA was extracted using the RNeasy Plus Mini Kit (Qiagen, Hilden, Germany, cat # 74104) according to the manufacturer’s instructions. The RNA quantity and quality were evaluated using the Nanodrop 8000 spectrophotometer (Thermo Fisher Scientific, Waltham, MA, USA). Subsequently, 2 µg of total RNA were reverse transcribed into cDNA using the iScript^TM^ cDNA Synthesis Kit (Bio-Rad, Hercules, CA, USA, cat # 1708841) and the Bio-Rad MyCycler thermal cycler (Bio-Rad, Hercules, CA, USA). Gene expression profiles of SAP family members, the EAP1 adhesion gene, pro-inflammatory cytokines, and human β-defensins (hBD-1 to hBD-4) were evaluated by real-time PCR using gene-specific primers (Table 1 and Table 2) with the iTaq^TM^ Universal SYBR^®^ Green Supermix (Bio-Rad Laboratories, Hercules, CA, USA; cat. #1725124). ACT1 and GAPDH were used as housekeeping genes for fungal and human cells, respectively. Relative gene expression levels were calculated using the 2^−∆∆Ct^ method. Three technical replicates and four biological replicates were performed.

### 4.15. Adhesion of C. albicans to Gingival Epithelial Cells

*C. albicans* cells were either pretreated or not with *Viroelixir* at a dilution of 1/50 for 24 h prior to co-culture. Subsequently, 2 × 10^6^ fungal cells were added to 6-well plates containing 5 × 10^5^ Ca9-22 epithelial cells per well. Ca9-22 cells were seeded 24 h prior to infection in antibiotic- and antifungal-free medium. After 6 and 24 h of co-culture, cells were washed three times with phosphate-buffered saline (PBS) to remove non-adherent *C. albicans*, and adherent cells were stained with crystal violet as previously described. Images were acquired using a Nikon Eclipse TS100 optical microscope at 20× magnification. A higher *Viroelixir* concentration (1/50) was selected for this assay to ensure a measurable effect on fungal adhesion to epithelial cells. All experiments were performed in at least four independent biological replicates, each conducted in triplicate.

### 4.16. ELISA Assay

A total of 3 × 10^5^ GMSMK cells were seeded in 6-well plates and co-cultured with 2 × 10^5^ *C. albicans* cells, pretreated or not with *Viroelixir* at dilutions of 1/1000 and 1/200 (*v*/*v* of the stock solution), for 24 h. Lower *Viroelixir* concentrations (1/1000 and 1/200) were selected in this assay to allow detection of subtle changes in host inflammatory responses without excessive perturbation of epithelial cells. Following co-culture, supernatants were collected, and pro-inflammatory cytokines and antimicrobial peptides were quantified using Thermo Fisher ELISA kits according to the manufacturer’s instructions: IL-6 (Cat# 88-7068-88), IL-8 (Cat# 88-8086-88), and hBD-2 (Cat# EK-072-37). Protein levels were normalized to 10^6^ cells. All experiments were performed in at least five independent biological replicates, each conducted in triplicate.

### 4.17. Signaling Pathways Study by Flow Cytometry

GMSMK cells (3 × 10^5^) were seeded in a 6-well plate with DMEM medium (Gibco, Thermo Fisher Scientific, Waltham, MA, USA; Cat# 11320033) supplemented with 10% FBS. After 24 h of adhesion, cells were serum-starved for 12 h. GMSMK cells were then incubated with 2 × 10^5^ *C. albicans* cells, pretreated for 24 h with varying dilutions of *Viroelixir* (1/1000 and 1/200 of *v*/*v* of the stock solution) or untreated *C. albicans*, at 37 °C in a 5% CO_2_ incubator for 60 min. After incubation, GMSMK cells were washed three times with PBS (1×) to remove *C. albicans* and were examined using a phase contrast microscope. Photographs were acquired using an epifluorescence light microscope (Axiophot, Zeiss, Oberkochen, Germany). Flow cytometric analysis was performed using an Accuri^TM^ C6 flow cytometer (Becton Dickinson, Franklin Lakes, NJ, USA). A minimum of 10,000 cells per sample were collected and analyzed. After washing with PBS containing 2% FBS, GMSMK cells were labeled with 5 µL of CD284 (TLR4) monoclonal antibody (HTA125), Alexa Fluor^TM^ 488 (eBioscience^TM^, San Diego, CA, USA, Cat# 53-9917-42) and incubated at room temperature in the dark for 60 min in PBS with 2% FBS. For NF-κB measurement, cells were fixed with 10% formaldehyde at room temperature for 20 min, centrifuged at 500× *g* for 5 min, and washed twice with PBS containing 2% FBS. Fixed cells were resuspended in 90% methanol and incubated at 4 °C for 20 min. Cells were then incubated with NF-κB p65 (F-6) monoclonal antibody (1:200) (Santa Cruz Biotechnology, Dallas, TX, USA, Cat# sc-8008) for 60 min at room temperature. After washing, cells were incubated in the dark with the secondary antibody Anti-Mouse IgG (H + L), F(ab’)_2_ Fragment (Alexa Fluor^®^ 488 Conjugate) (eBioscience^TM^, San Diego, CA, USA, Cat# 53-9890-42) (1:200) for 30 min in PBS + 2% FBS. Following the immunoreaction, cells were resuspended in PBS + 2% FBS, and fluorescence intensities of FITC were measured by flow cytometry. All experiments were performed in at least three independent experiments, each conducted in triplicate.

### 4.18. Statistical Analysis

All experiments were repeated at least three times, with experimental values presented as mean ± SEM. Differences between the controls and treatment conditions were determined using the one-way ANOVA or the two-way ANOVA tests. GraphPad PRISM 9.4.0 software was used for this purpose. PCR data were analyzed using the 2^−∆∆Ct^ relative expression method (Livak). Values * *p* < 0.05, ** *p* < 0.01, and *** *p* < 0.001 were considered statistically significant.

## 5. Conclusions

This study highlights that *Viroelixir* is associated with reduced *C. albicans* growth and modulation of several virulence-related traits, including biofilm formation, hyphal transition, and adhesion capacity. These changes were accompanied by alterations in host inflammatory responses, particularly in NF-κB activation and cytokine production in gingival epithelial cells.

The findings suggest that *Viroelixir* may influence host–pathogen interactions by modulating fungal behavior and the epithelial immune response. However, the underlying molecular mechanisms remain to be fully elucidated.

Overall, these results support the potential of natural antifungal compounds as complementary approaches for managing fungal infections. Further investigations using advanced models, including engineered oral mucosa or vivo systems, are required to confirm the translational relevance of these observations.

## Figures and Tables

**Table 1:** Primers sequences corresponding to the SAPs family, EAP1 and ACT1 fungal genes.

*C. albicans* Genes	Primer Sequence	Amp Size (bp)
EAP1	5′-CTGCTCACTCAACTTCAATTGTCG-3′ 3′-GAACACATCCACCTTCGGGA-5′	51
SAP1	5′-TTTCATCGCTCTTGCTATTGCTT-3′ 3′TGACATCAAAGTCTAAAGTGACAAAACC-5′	86
SAP2	5′-TCCTGATGTTAATGTTGATTGTCAAG-3′ 3′-TGGATCATATGTCCCCTTTTGTT-5′	82
SAP3	5′GGACCAGTAACATTTTTATGAGTTTTGAT-3′ 3′-TGCTACTCCAACAACTTTCAACAAT-5′	87
SAP4	5′-AGATATTGAGCCCACAGAAATTCC-3′ 3′-CAATTTAACTGCAACAGGTCCTCTT-5′	81
SAP5	5′-CATTGTGCAAAGTAACTGCAACAG-3′ 3′-CAGAATTTCCCGTCGATGAGA-5′	77
SAP6	5′-CCTTTATGAGCACTAGTAGACCAAACG-3′ 3′-TTACGCAAAAGGTAACTTGTATCAAGA-5′	101
SAP7	5′-GAAATGCAAAGAGTATTAGAGTTATTAC 3′GAATGATTTGGTTTACATCATCTTCAACTG-5′	196
SAP8	5′-TCCCTGAAGACATTGATAAAAGAGC-3′ 3′-AGAATCAACCACCCATAAATCAGAA-5′	278
SAP9	5′-ATTTACTCCACAGTTTATCACTGAAGGT-3′ 3′-CCACAAGAACCACCCTCAGTT-5′	86
ACT1	5′-GCTGGTAGAGACTTGACCAACCA-3′ 3′-GACAATTTCTCTTTCAGCACTAGTAGTGA-5′	87

**Table 2:** Primers sequences used for qRT-PCR to evaluate the expression of pro- inflammatory cytokines and β-defensin genes in human gingival epithelial cells.

Human Genes	Primer Sequence	Amp Size (bp)
IL-1β	5′-CTGTCCTGCGTGTTGAAAGA-3′ 3′-TTGGGTAATTTTTGGGATCTACA-5′	69
IL-6	5′-TCTCCACAAGCGCCTTCG-3′ 3′-CTCAGGGCTGAGATGCCG-5′	203
IL-8	5′-TTGGCAGCCTTCCTGATT-3′ 3′-AACTTCTCCACAACCCTCTG-5′	248
hBD-1	5′-GCCTCTCCCCAGTTCCTGAA-3′ 3′-GCAGAGAGTAAACAGCAGAAGGTA-5′	82
hBD-2	5′-TGTGGTCTCCCTGGAACAAAAT-3′ 3′-GTCGCACGTCTCTGATGAGG-5′	105
hBD-3	5′-CTTCTGTTTGCTTTGCTCTTCCT-3′ 3′-CTGTTCCTCCTTTGGAAGGCA-5′	138
hBD-4	5′-CACTCTACCAACACGCACCTAG-3′ 3′-CGCAACTGGAACCACACACT-5′	133
GAPDH	5′-GGTATCGTCGAAGGACTCATGAC-3′ 3′-ATGCCAGTGAGCTTCCCGTTCAGC-5′	180

## Data Availability

All data generated or analyzed during this study are included in this published article.

## Supplementary Materials

The following supporting information can be downloaded at: https://www.mdpi.com/article/10.3390/antibiotics15040420/s1, Figures S1–S4. (A) IL-1 expression at mRNA level after stimulation with *Viroelixir*, (B) hBD-1 expression. (C) hBD-3 and (D) hBD-4 *mRNA* expression levels in GMSMK cells following co-culture with *C. albicans* whether pre-exposed or not to Viroelixir at 1/1000 and 1/200. mRNA expression was analyzed by qRT-PCR and normalized to GAPDH. Data are presented as mean ± SEM. ** *p* < 0.001, and *** *p* < 0.0001 are considered as statistically significant.

## Abbreviations

*C. albicans*	*Candida albicans*
*Ampho-B*	*Amphotericin B*
OD	Optical Density
ELISA	Enzyme-Linked Immunosorbent Assay
EAP1	Epithelial Adhesin Protein 1
GMSMK	Gingival Stromal Mesenchymal Cells
hBD	Human Beta-Defensin
IC50	Half Maximal Inhibitory Concentration
MIC	Minimum inhibitory concentration
MTT	3-(4,5-Dimethylthiazol-2-yl)-2,5-Diphenyltetrazolium Bromide
NF-κB	Nuclear Factor Kappa-Light-Chain-Enhancer of Activated B Cells
PKC	Protein Kinase C
ROS	Reactive Oxygen Species
RT-PCR	Reverse Transcription Polymerase Chain Reaction
SAP	Secreted Aspartyl Protease
TLR	Toll-Like Receptor
TNF-α	Tumor Necrosis Factor Alpha
HWP1	Hyphal Wall Protein 1
p38 MAPK	p38 Mitogen-Activated Protein Kinases
JNK	c-Jun N-terminal Kinase
IL-6, IL-8, IL-1β	Interleukin-6, Interleukin-8, Interleukin-1 Beta
RPMI	Roswell Park Memorial Institute Medium
DMSO	Dimethyl Sulfoxide
FBS	Fetal Bovine Serum
SEM	Scanning Electron Microscopy
PBS	Phosphate-Buffered Saline
DMEM	Dulbecco’s Modified Eagle Medium
FITC	Fluorescein Isothiocyanate
RNA	Ribonucleic Acid
DNA	Deoxyribonucleic Acid
cDNA	Complementary DNA
PCR	Polymerase Chain Reaction
Ct	Cycle Threshold
bp	Base Pair
PI	Propidium Iodide
MEC	Minimal effective concentration
