# Supplementary material for: Evaluation of the Antifungal Activity of the Polyphenol Formulation Viroelixir Against Candida albicans
Source: Antibiotics (Basel). 2026 Apr 21;15(4):420. doi: 10.3390/antibiotics15040420 (PMC13113709; doi:10.3390/antibiotics15040420)
Supplement: Supplementary file 1 [file antibiotics-15-00420-s001.zip › antibiotics-4147323-supplementary.pdf]

antibiotics

MDPI

Supplementary data

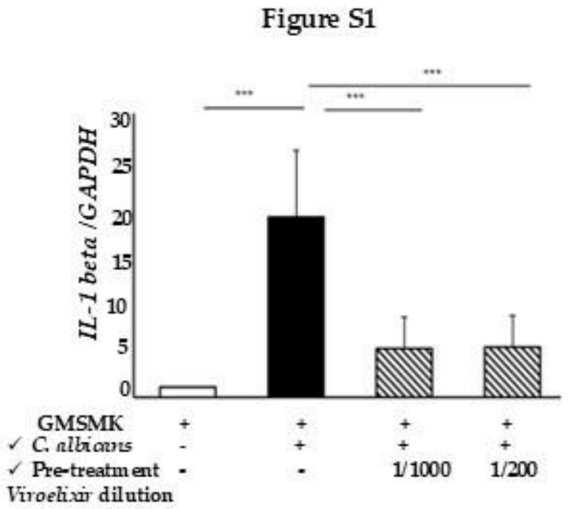

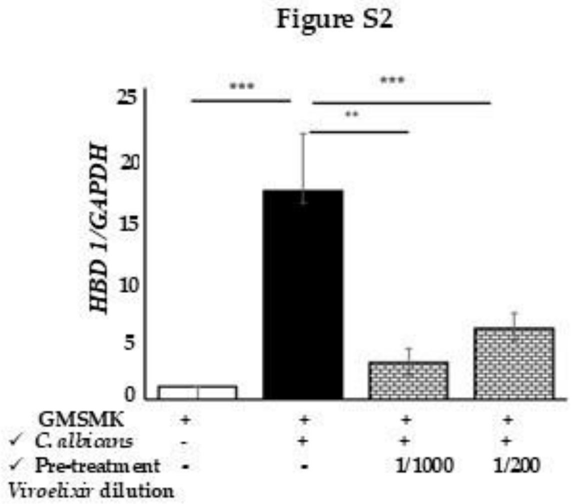

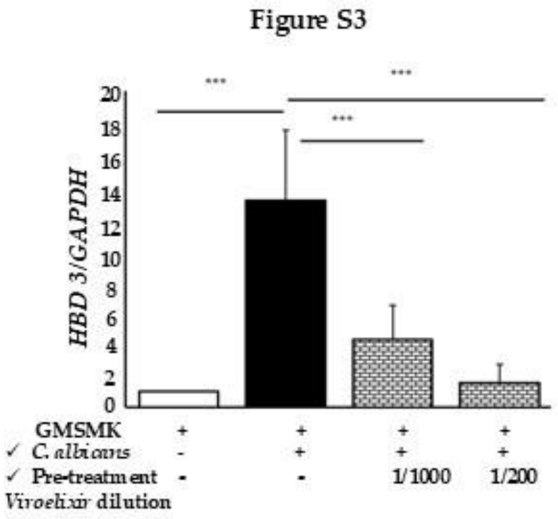

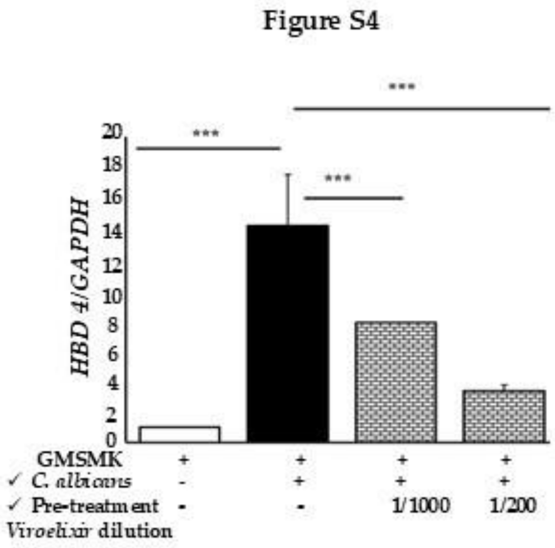

Figure S1 to S4. A) IL-1 expression at mRNA level after stimulation with *Viroelixir*, B) hBD-1 expression. C) hBD-3 and 4) hBD-4 *mRNA* expression levels in GMSMK cells following co-culture with *C. albicans* whether pre-exposed or not to Viroelixir at 1/1000 and 1/200. mRNA expression was analyzed by qRT-PCR and normalized to GAPDH. Data are presented as mean ± SEM. ** $p < 0.001$, and *** $p < 0.0001$ are considered as statistically significant

 https://doi.org/10.3390/antibiotics15040420
